# Selective apoptotic cell death effects of oral cancer cells treated with destruxin B

**DOI:** 10.1186/1472-6882-14-207

**Published:** 2014-06-28

**Authors:** Rosa Huang Liu, Shih-Pin Chen, Tsong-Ming Lu, Wei-Yu Tsai, Chung-Hung Tsai, Chi-Chiang Yang, Yew-Min Tzeng

**Affiliations:** 1 School of Nutrition, Chung Shan Medical University, Taichung, Taiwan; 2 Institute of Medicine, Chung Shan Medical University, Taichung, Taiwan; 3 School of Medicine, Chung Shan Medical University, Taichung, Taiwan; 4 Division of Pulmonary Medicine, Department of Internal Medicine, Chung Shan Medical University Hospital, Taichung, Taiwan; 5 Department of Neurology, Chung Shan Medical University Hospital, Taichung, Taiwan; 6 School of Medical Laboratory and Biotechnology, Chung Shan Medical University, 110, Section 1, Chien-Kuo North Road, Taichung 40201, Taiwan; 7 Department of Pathology, Chung Shan Medical University Hospital, Chung Shan Medical University, Taichung, Taiwan; 8 Department of Clinical Laboratory, Chung Shan Medical University Hospital, Taichung, Taiwan; 9 Institute of Biochemical Sciences and Technology, Chaoyang University of Technology, 168 Jifong East Road, Wufong District, Taichung 41349, Taiwan

**Keywords:** Destruxin, Cytotoxicity, Oral cancer, Apoptosis

## Abstract

**Background:**

Recent studies have revealed that destruxins (Dtx) have potent cytotoxic activities on individual cancer cells, however, data on oral cancer cells especial human are absent.

**Methods:**

Destruxin B (DB) was isolated and used to evaluate the selective cytotoxicity with human oral cancer cell lines, GNM (Neck metastasis of gingival carcinoma) and TSCCa (Tongue squamous cell carcinoma) cells, and normal gingival fibroblasts (GF) were also included as controls. Cells were tested with different concentrations of DB for 24, 48, and 72 h by MTT assay. Moreover, the mechanism of cytotoxicity was investigated using caspase-3 Immunofluorescence, annexin V/PI staining, and the expression of caspase-3, Bax, and Bcl-2 by western blotting after treated with different concentrations of DB for 72 h as parameters for apoptosis analyses.

**Results:**

The results show that DB exhibited significant (*p* < 0.01) and selective time- and dose-dependent inhibitory effects on GNM and TSCCa cells viability but not on GF cells. The data suggested that DB is capable to induce tumor specific growth inhibition in oral GNM and TSCCa cancer cells *via* Bax/Bcl-2-mediated intrinsic mitochondrial apoptotic pathway in time- and dose-dependent manners.

**Conclusions:**

This is the first report on the anti-proliferation effect of DB in oral cancer cells. The results reported here may offer further evidences to the development of DB as a potential complementary chemotherapeutic target for oral cancer complications.

## Background

Destruxins (Dtx) are the most abundant secondary metabolites of the entomopathogenic fungus *Metarhizium anisopliae* and usually secreted into the culture medium during growth [1]. Destruxins, especially Destruxin A, B and E (DA, DB, and DE), are a class of insecticidal cyclic depsipeptides [2]. Previous studies have also shown destruxins exhibited strong biological effects; for example, destruxins disturbs macromolecular syntheses (DNA, RNA and protein synthesis) [3], produces anti-hepatitis B effects [4-6] and modifies the DNA content of murine leukemia cells [7-9]*in vitro*. Recently, Dtx came into focus of interest as an anticancer therapeutics [10-13]. Our previous data revealed a significant inhibition of colorectal cancer cell (HT-29) viability was observed with DB treatment in time- and dose-dependent manners [10]. Moreover, DB administered subcutaneously daily at 0.6-15 mg/kg was safe and effective in inhibiting the growth of colorectal cancer cells in a murine xenograft animal model. Expression of Bax, cleaved poly (ADP-ribose) polymerase and active caspase-3 were observed with DB treatment and the increase in tumor volumes of treated groups were significantly (P < 0.05) less than those of the mock-treated group. Therefore DB was suggested to have the potential as a new therapeutic agent against human colorectal cancer.

Oral cancer, a subtype of head and neck cancer, may arise as a primary lesion originating in the oral tissues that line the mouth, lips, gingiva (gums) and tongue. Around 90% are squamous cell carcinomas. In the battle against cancer, one of the main technologies used for treatment is chemotherapy. Chemotherapy is the use of chemicals. Chemotherapy works by interfering with the cancer cell’s ability to grow or destroying the cancer cells. Biological agent, such as cetuximab has recently been shown to be effective in the treatment of squamous cell head and neck cancers, and is likely to has an increasing role in the future management of this condition when used in conjunction with other established treatment modalities [14]. However, both mortality and morbidity of patients with oral cancer have not significantly much improved in the past decades [15]. New chemicals against oral cancer are desperate in needs.

Previously, we have demonstrated some chemicals including caffeic acid phenethyl ester analogues [16], Methylantcinate A [17], and derivatives from *Antrodia camphorate*[18] exhibit significant anti-oral cancer activities. Our previous data also revealed a significant inhibition of colorectal cancer cell viability was observed with DB treatment both *in vitro* and *in vivo*[10]. Although recent studies have revealed that Dtx have potent cytotoxic activities on individual cancer cells [7-13], data on oral cancer cells are absent. Consequently, this is the first study aimed to investigate the impact of DB on human oral cancer cells *in vitro* growth and survival, as well as with special focus on the apoptotic cell death pathway. In this study, DB was isolated and used to evaluate the selective cytotoxicity with human oral cancer cell lines, GNM (Neck metastasis of gingival carcinoma) and TSCCa (Tongue squamous cell carcinoma) cells, and normal gingival fibroblasts (GF) were also included as controls. Hopefully, together with previous findings, we could evaluate different aspects of different cancer cells and molecular biological characteristics and assess potential novel cancer treatment regimens of DB.

## Methods

### Production of destruxins

A culture of *M. anisopliae* F061 *var. anisopliae* kindly provided by Dr. Suey-Sheng Kao, Taiwan Agricultural Chemicals and Toxic Research Institute (Wufeng, Taiwan), was used in this study. The culture method was used as described previously [19]. Briefly, the spore suspension culture from -80°C was thawed at room temperature and inoculated into a 500-ml Erlenmeyer flask with a baffle containing 200 ml of 3% (w/v) Czapek-Dox (CD) broth (BD, Spark, MD, USA) and 0.75% bacto-peptone (BD, Spark, MD, USA) as seed culture. The flask was cultivated in an incubator (LM-575R, Yih-Der Co., Taipei, Taiwan) at 200 rpm, 28°C for 4 days. For the stirred-tank cultivation, the inoculum (10% of the working volume) was transferred from the flask of the 4 day old seed culture to the reactor, which contained 3 L of the desired medium. Cultivations were conducted in a 5 L stirred tank reactor (BTF-A5L, Bio-Top Inc, Taichung, Taiwan) at 28°Cwith the aeration rate regulated at 0.3 vvm (volume air/volume liquid/min). The culture medium (pH 9.0) was maintained by automatic addition of 2 N NaOH or 1 N HCl at a agitation rate of 150 rpm. After 14 days, the fermentation broth was harvested and purified as the following procedures.

### Purification of destruxins

The destruxins were isolated and purified according to the method of Chen et al. [20]. The culture medium was harvested after incubation for 14 days and centrifuged at 9000 rpm for 20 min. The supernatant was adjusted to pH 4.0 by 1 N HCl then extracted with ethyl acetate (sample: EA = 5:2, v/v), and the organic phase was evaporated with a rotary vacuum evaporator (model N-1, Eyela, Tokyo, Japan) at 45°C. The concentrate was diluted with 2 times volume of acetonitrile and filtered through a 0.22 μm chromatodisc unit before HPLC analysis. The sample (800 μ L) was injected into a preparative column (Cosmosil 15 C18-AR-II column, 28 × 250 mm, 15 μm). The eluent from the column was monitored at 215 nm with a L-7100 pump and a L-7400 UV detector (Hitachi, Tokyo, Japan). The mobile phase was: 80% Methanol/H_2_O. The eluting solvent was set at 10 mL/min. Fractionated samples were characterized by analytic HPLC, ESI-MASS and ^1^H NMR spectroscopes.

### Cell culture

The GNM, TSCCa, and GF cells used in this study have been reported previously [16-18,21]. Briefly, GNM cells were in RPMI 1640 with 10% supplemented with 10% fetal bovine serum (FBS; Life Technologies, Carlsbad, CA, USA). TSCCa and GF cells were grown in Dulbecco’s modified Eagle’s medium (DMEM; Life Technologies, Carlsbad, CA, USA) supplemented with 10% FBS (Life Technologies, Carlsbad, CA, USA). Both medium were added with penicillin (100 IU/ml) and streptomycin (100 μg/ml). Briefly, the cells were maintained in the appropriate growth medium at 37°C in a humidified atmosphere of 5% CO_2_ and 95% air and used over a restricted culture period of 10 passages.

### Cell viability

The colorimetric MTT (3-(4,5-dimethylthiazol-2-yl)-2,5-diphenyl tetrazolium bromide, tetrazolium) assay will be used to observe the survival ratio of cells which has been used in our lab before [16,21-24]. Cells will be seeded in 96-wells plates in triplicate in medium containing 2% (V/V) serum. After 24 h, 20 μl MTT solution (5 mg/ml) were added to each well of cells group and then incubated 4 h in a 5% CO_2_ incubator 37°C. After the reaction was finished, the supernatant was discarded and 200 μl DMSO (dimethyl sulfoxide) was added to each well. A blank control which contained medium and MTT, but no cells, was also included. Cell growth and growth inhibition will be measured at 570 nm with a spectrophotometer (Model 550, BIO-RAD, Palo Alto, California, USA).

### Immunofluorescence

This is manipulated as described before [21,23]. Briefly, cells were incubated and grown as a monolayer in chamber slides at 37°C in humidified 5% CO_2_/95% air atmosphere. After treatment, cells were rinsed twice in phosphate-buffered saline (PBS, pH 7.4), immediately fixed with ice-cold acetone at -20°C for 20 min. After 3 washes with PBS, cells were incubated with 10% normal goat serum (NGS) in PBS for 1 h and then incubated with 1% NGS in PBS for 10 min to block nonspecific binding sites. Two-hundred microliters of rabbit polyclonal anti-caspase-3 primary IgG antibody (Clone H-277, Santa Cruz Biotechnology, Inc., Dallas, Texas, USA) diluted 1:100 in PBS-10% FCS was added to each well for 45 min at 37°C. After washing three times in PBS, 200 μl of secondary goat anti-rabbit IgG-FITC (fluorescein isothiocyanate) conjugated antibody (sc-2012, Santa Cruz Biotechnology, Inc., Dallas, Texas, USA) was diluted 1:200 in PBS-10% FBS and then added to each well for 45 min at 37°C. After washing three times in PBS, cells were visualized using a fluorescent microscope.

### Annexin V-fluorescein isothiocyanate (FITC) and propidium iodide (PI) staining

Annexin V binding was assayed by fluorescence-activated cell sorter (FACS) analysis according to the ApoAlert Annexin V protocol (BD Biosciences Clontech Palo Alto, CA, USA) as previously described [17,21,23,24]. Briefly, the tested cells were washed with PBS, detached from the culture plates with 0.25% trypsin and gently washed once with the growth medium. Then 5 × 10^5^ cells were washed once and resuspended in 200 μL of the 1X binding buffer, mixed with 5 μL of annexin V-FITC and 10 μL of PI, and incubated at room temperature for 5 min in the dark. Then the reaction volume was adjusted with the binding buffer to 500 μL. The cells were subjected to FACS analysis in a flow cytometer (Becton Dickinson, San Jose, CA) using a single laser emitting excitation light at 488 nm.

### Sodium dodecyl sulfate (SDS)-polyacrylamide gel electrophoresis (PAGE) and western blotting

SDS-PAGE [25-27] and western blotting were performed as previously described [17,24]. Total proteins were solubilized and extracted from tissue with 300 μl lysis buffer. The lysates were used to estimate the protein content with Bradford protein assay. Equal amounts of protein (50 μg) from each sample were then subjected to electrophoresis on a 12% (v/v) SDS-polyacrylamide gel. After electrophoresis, proteins were electroblotted to a PolyScreen^®^ PVDF hybridization transfer membrane (PerkinElmer, Boston, MA, USA). The membrane was blocked at room temperature with blast blocking buffer [1% blast blocking reagent (PerkinElmer, Boston, MA, USA) in Tris buffered saline (TBS) containing 0.05% (v/v) Tween (TBS-T)]. Then the membrane was washed three times with TBS-T and incubated overnight at 4°C with the primary antibody, mouse monoclonal anti-caspases-3 (1:1000, v/v), or rabbit monoclonal antibodies to poly (ADP-ribose) polymerase (PARP) (1:4000, v/v), Bcl-2-associated X protein (Bax) (1:8000, v/v) and β-actin (1:2000, v/v), followed by 1 h incubation with a 1:10000 (v/v) dilution of the appropriate horseradish peroxidase (HRP)-conjugated secondary antibody. Primary antibodies (β-actin, PARP, Bax) were purchased from Cell Signaling Technology Inc. (Danvers, MA, USA), whereas secondary antibodies (anti-rabbit IgG and anti-mouse IgG HRP-labeled) were purchased from PerkinElmer (Boston, MA, USA). After incubation, the membrane was washed with TBS-T three times, and the antigen-antibody complexes were visualized by the enhanced chemiluminescence system (PerkinElmer). The relative protein expression with the control defined as 100% was measured by Image J software (National Institutes of Health, USA).

### Statistics analysis

The Kruskal-Wallis test of analysis in SPSS (version 10.0) software will be used to study the significant change between different cells in the experiments used in this study. The *p* value < 0.05 will be taken to be regarded as significant.

## Results

### Selective cytotoxicity of DB for oral squamous cell carcinoma cell lines

**Figure 1:**
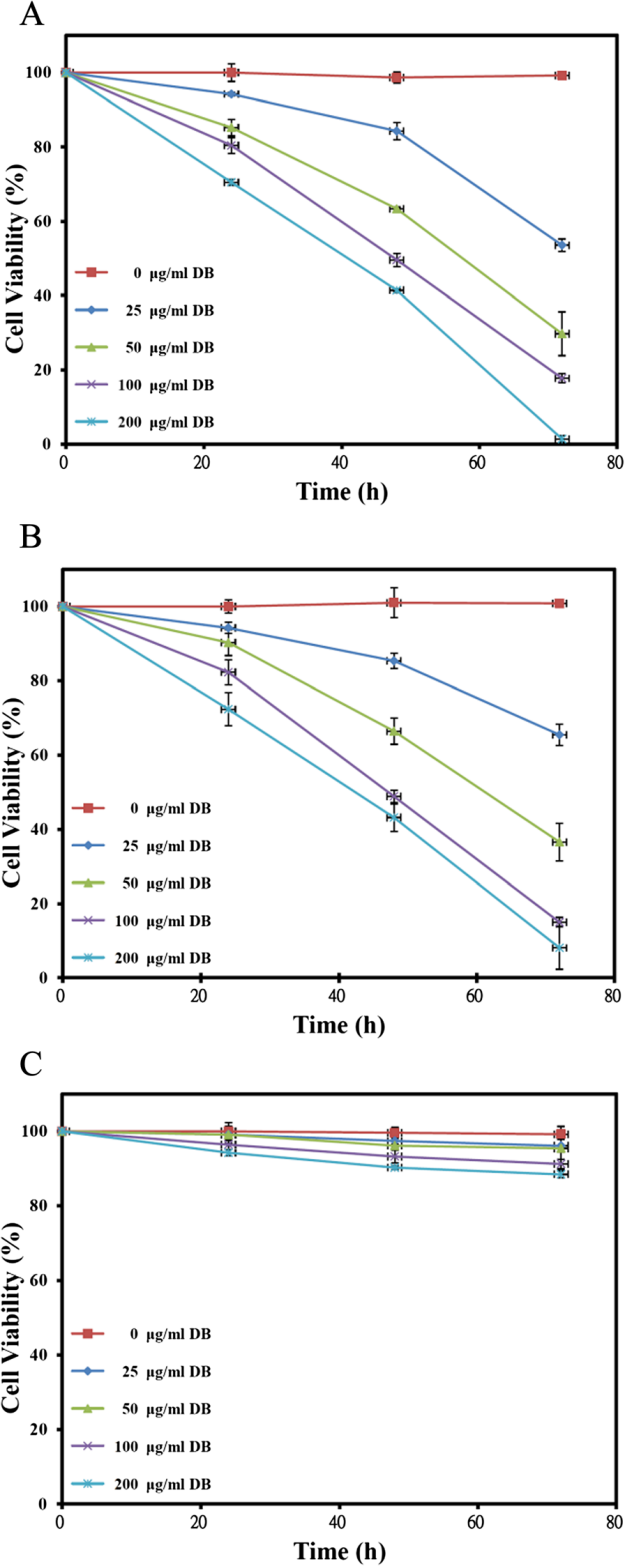
(A) GNM, (B) TSCCa, and (C) GF cells viability treated with different concentrations of DB for 24, 48, and 72 h.

**Figure 2:**
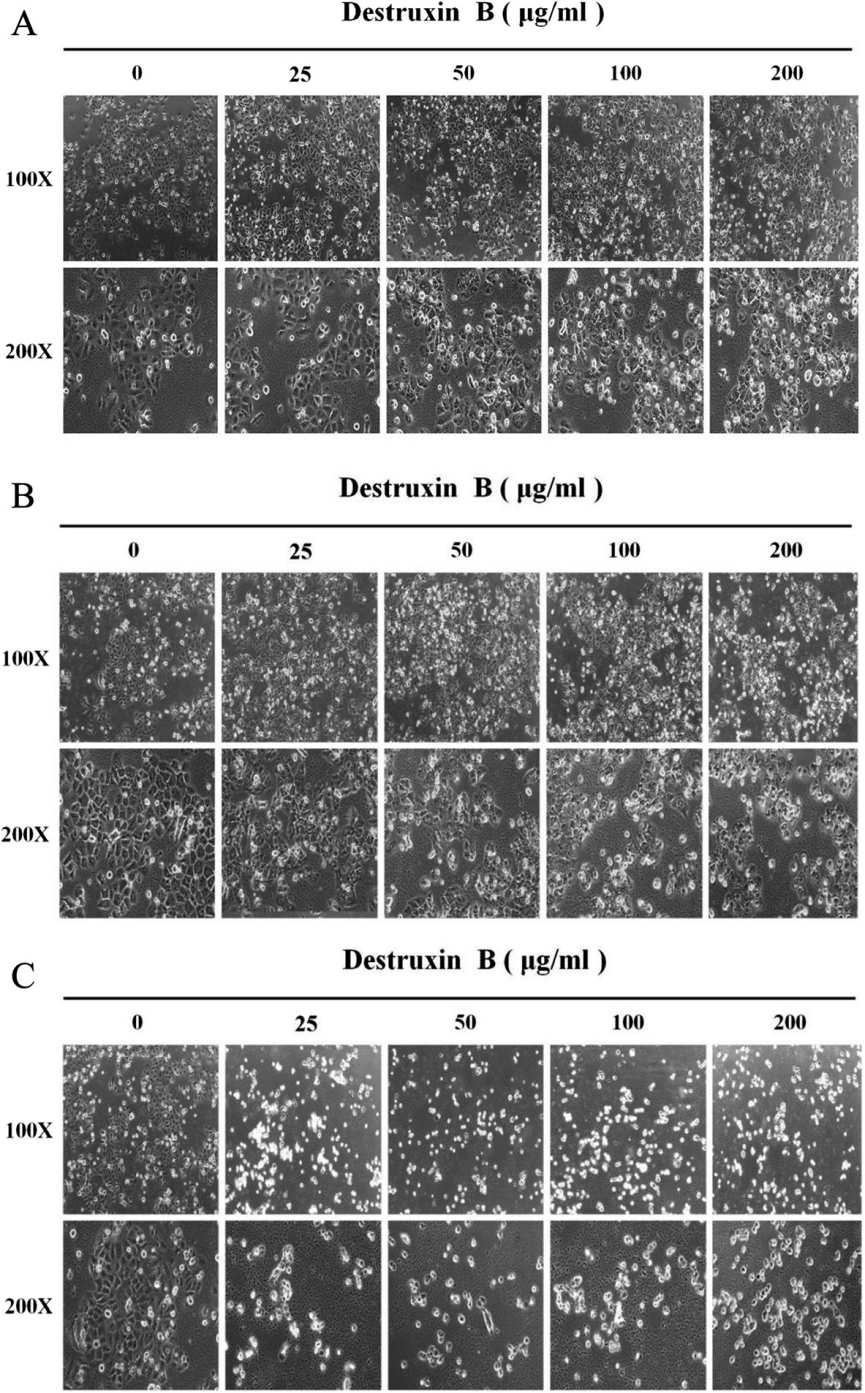
The cell morphology of GNM treated with different concentrations of DB for (A) 24, (B) 48, and (C) 72 h under a microscope (100X and 200X).

**Figure 3:**
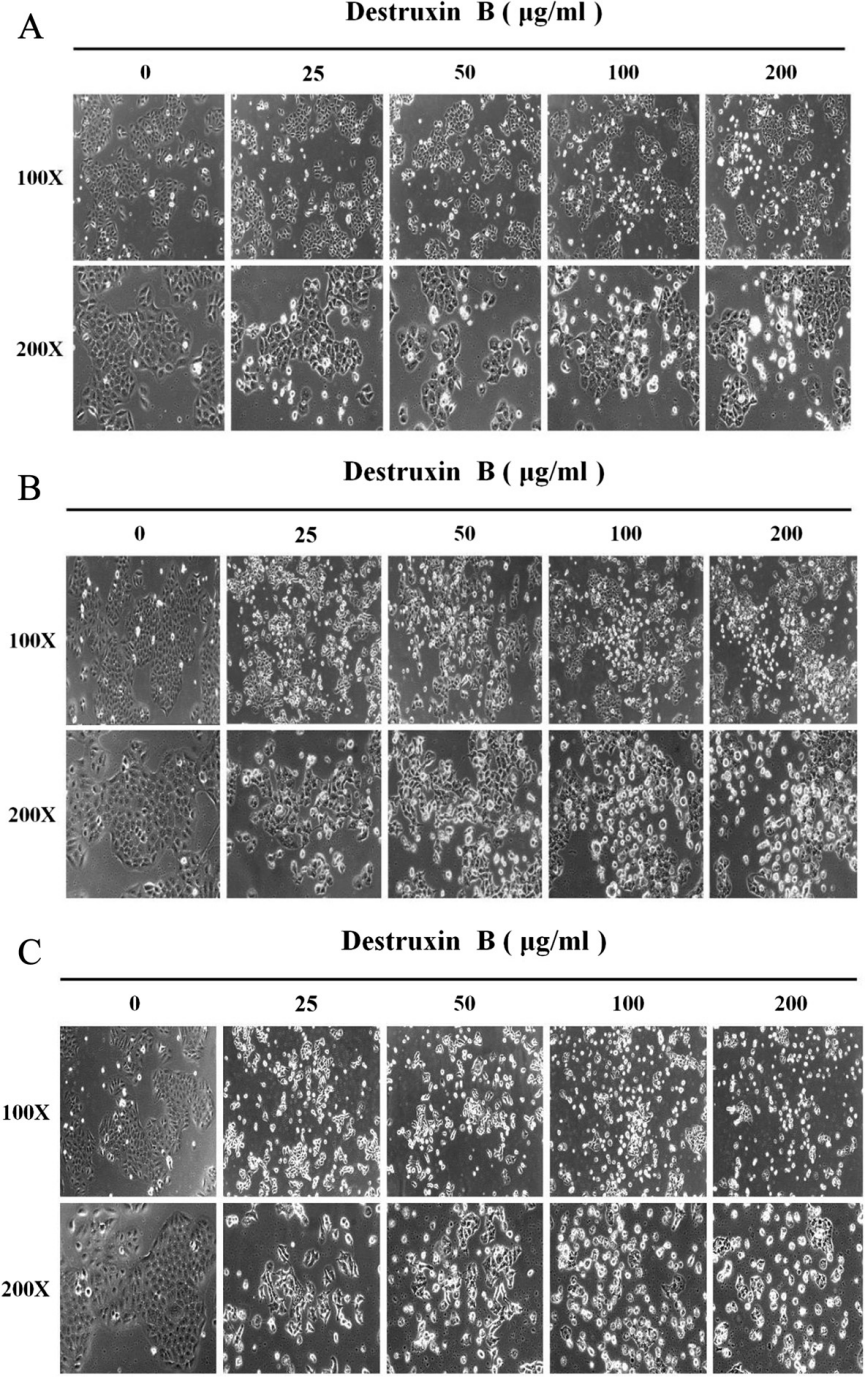
The cell morphology of TSCCa treated with different concentrations of DB for (A) 24, (B) 48, and (C) 72 h under a microscope (100X and 200X).

The isolation, purification, and identification of DB in this lab have been reported before [10]. The selective cytotoxicity of DB was examined in human oral squamous cell carcinoma cell lines GNM (Neck metastasis of gingival carcinoma) and TSCCa (Tongue squamous cell carcinoma) cells, as well as the normal gingival fibroblast (GF) cells. Aforementioned three cell lines were treated with various concentrations of DB for 24, 48, and 72 h, and the viable cells were measured by the 3-(4,5-dimethylthiazol-2-yl)-2,5-diphenyl-2H-tetrazolium bromide (MTT) assay (Figure 1). The results showed that DB exhibited significant (P < 0.05) time- and dose-dependent inhibitory effects on GNM and TSCCa cells viability. The concentrations of DB that result in a 50% reduction in absorbance (IC_50_) for GNM cells after 24, 48, and 72 h treatment were 281.9, 84.7 and 31.2 μg/ml, respectively, and were 289.4, 86.5, and 38.3 μg/ml, respectively, for TSCCa cells (Table 1). The cytotoxic effects of DB were consistent in these two cell lines with nearly equal sensitivities, however, the effects in GNM cells is slightly superior than that in TSCCa cells. Interestingly, the proliferation of normal GF cells was only slightly reduced (88.4%) by DB treatment at the maximum concentration tested (200 μM) for 72 h suggesting a selective cytotoxic activity against tumor cells. The GNM and TSCCa cell microscopic morphology which treated with different concentrations of DB for 24, 48, and 72 h were also recorded to show the cytotoxicity (Figures 2 and 3, respectively).

**Table 1:** **The IC**_**50 **_**concentration of DB for GNM and TSCCa cells after 24, 48, and 72 h treatment**

**Cell**	**IC**_**50 **_**(μg/ml)**		
	**24 h**	**48 h**	**72 h**
GNM	281.9	84.7	31.2
TSCCa	289.4	86.5	38.3

### The mechanism of DB-induced cell death

**Figure 4:**
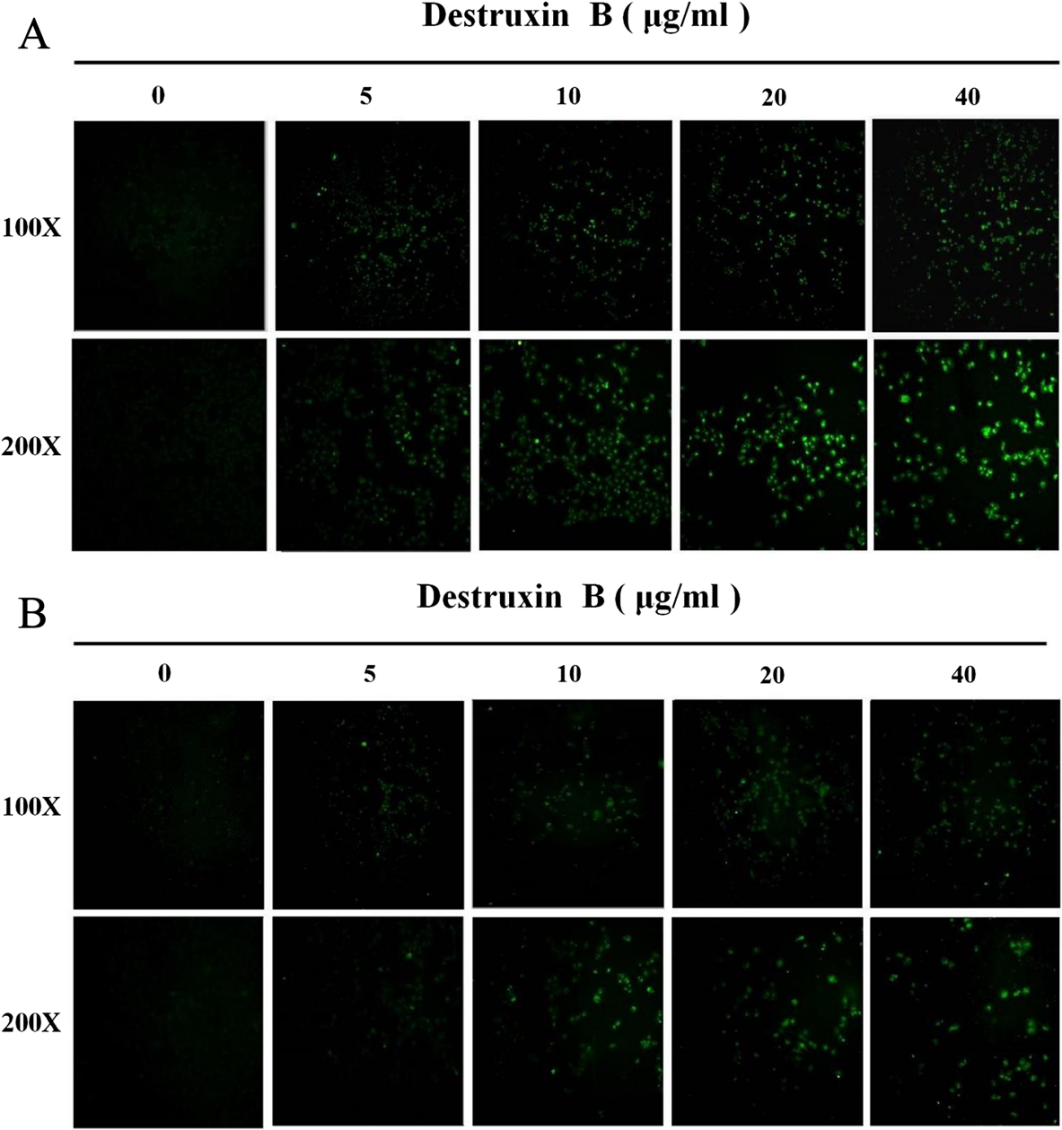
The (A) GNM and (B) TSCCa cells immunoflourecent labelled with anti-caspase-3 antibody for caspase-3 detection after treated with different concentrations of DB for 72 h.

**Figure 5:**
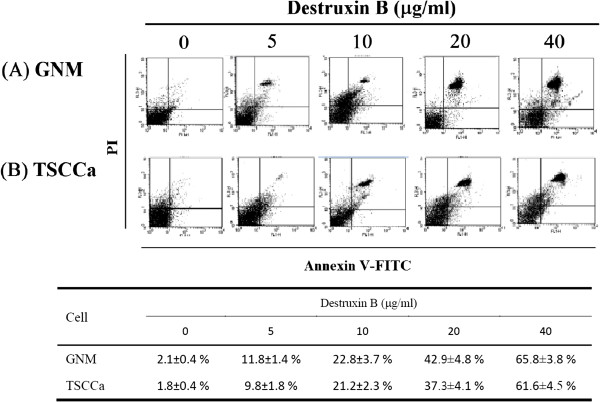
**The FACS analysis of (A) GNM and (B) TSCCa cells staining with annexin V-FITC and PI after treated with different concentrations of DB for 72 h.** The values of mean ± S.D. from triplicate experiments were also shown. The percentage indicated the population of cells with annexin V-FITC/PI double positive signals.

Several assays were used to examine the mechanism of DB-induced cell death. Among the caspases characterized in humans, caspase-3 is the main downstream effecter caspase that play essential roles in degrading the majority of key cellular components in apoptotic cells [28]. We therefore investigated the possible involvement of caspase-3 in DB-induced apoptosis. As both the IC50 of 72 h DB treatment for GNM and TSCCa cells were below 40 μg/ml (Figure 1), 40 μg/ml, used as the maximum concentration, and a series of two-fold dilutions (5, 10, and 20 μg/ml) of DB were designed for further analyses. For the apoptosis analyses, the GNM and TSCCa cells were immunofluorescence labelled with anti-caspase-3 antibody for caspase-3 detection after treated with 5, 10, 20, and 40 μg/ml of DB for 72 h (Figure 4A and B, respectively). The intensity of caspase-3 was demonstrated to be dose-dependent obviously. Furthermore, apoptosis is accompanied by loss of organization in the plasma membrane, and a marker of this event is the translocation of the phospholipid phosphatidylserine (PS) from the inner to the outer portion of the membrane [29]. Annexin V-FITC is a PS-binding protein that can be conjugated to fluorescent groups and used in conjunction with propidium iodide (PI), a fluorescent molecule that is impermeable to cells with intact membranes but permeable to dead cells in FACS analysis. In this study, FACS analysis of untreated GNM and TSCCa cells, the majority of cells appeared negative for staining with annexin V-FITC and PI (left, lower), indicating that the cells were healthy (Figure 5). Upon the treatment with different concentrations of DB for 72 h, FACS analysis showed significant shifts in the cell population. The percentage of apoptotic cells increased significantly with increasing DB treatment at 5, 10, 20 and 40 μg/ml for both the GNM and TSCCa cells (P < 0.05) (Figure 5A and B, respectively). The mean ± S.D. values from triplicate experiments with annexin V-FITC/PI double positive signals (right, upper) were also shown.

**Figure 6:**
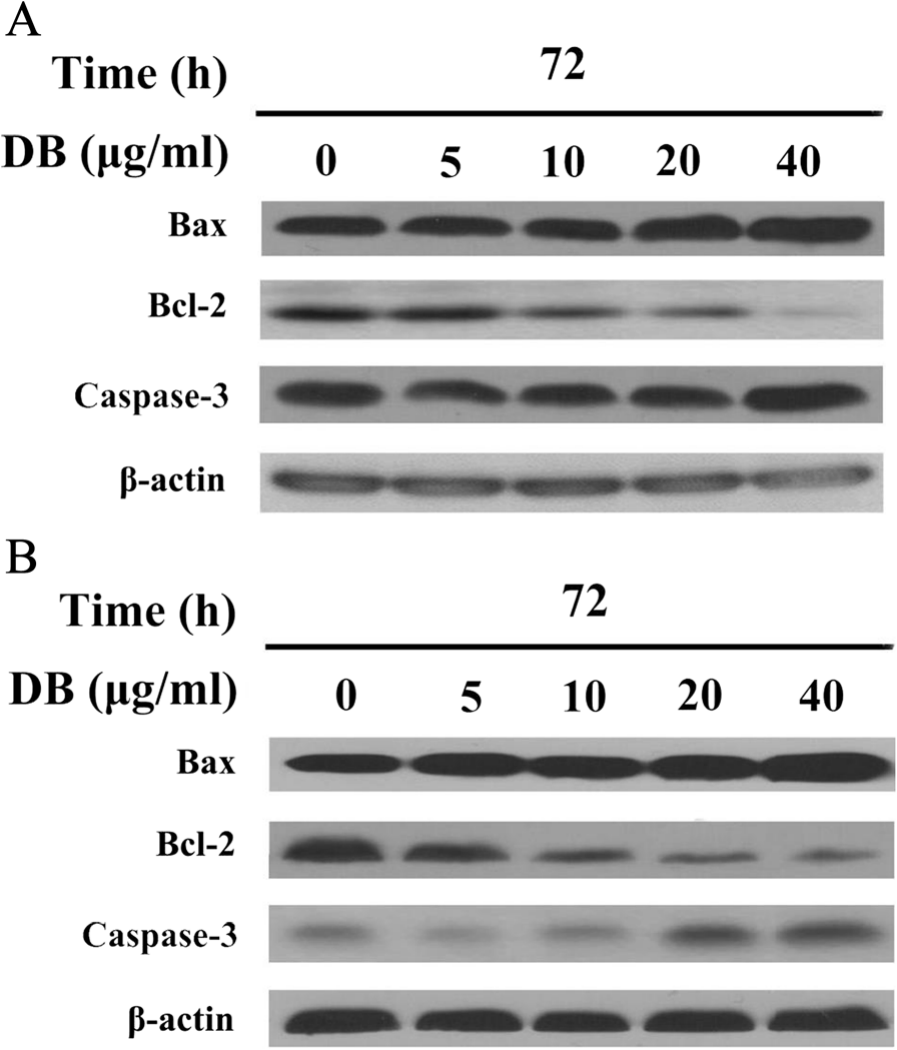
The (A) GNM and (B) TSCCa cells expression of caspase-3, Bax, and Bcl-2 detected by western blotting after treated with different concentrations of DB for 72 h.

To identify the molecular mechanism by which DB induces apoptosis, we further examined the levels of apoptosis-regulatory proteins in DB treated GNM and TSCCA cells using western blotting. The activation of caspase-3 and the presence of additional apoptotic indicators were examined for both the cell lines under conditions where the cells were treated with different concentration of DB. Treatment of GNM and TSCCA cells with DB for 72 h increased caspase expression dose-dependently which is another indicator of apoptosis. The anti-apoptotic protein Bcl-2 acts to prevent permeabilization of the outer mitochondrial membrane by inhibiting the action of the pro-apoptotic protein Bax [30]. Our results from western blot analysis showed that the treatment of GNM and TSCCA cells with DB for 72 h result the increased expression of pro-apoptotic Bax protein and decreased expression of anit-apoptotic Bcl-2 protein in a dose-dependent manner (Figure 6A and B, respectively). This finding suggested that the effect of the *Bax* gene product *via* the mitochondria [29] might be responsible for the modulation of DB-induced apoptosis in GNM and TSCCA cells. These data, the increased expression of pro-apoptotic Bax protein and decreased expression of anit-apoptotic Bcl-2 protein in a dose-dependent manner, are consistent with our previous findings [10,13] to suggest DB might exert pro-apoptotic effects through a Bax/Bcl-2-mediated mitochondrial apoptotic pathway.

## Discussion

Oral squamous cell carcinoma, which arises from the mucosa lining, accounts for over 90% of oral malignancies [31]. In addition, squamous cell carcinoma of the oral cavity is the 11th most common global cancer, accounting for 3% of all newly diagnosed cancer cases [32,33]; it is also the eighth most predominant cancer in males [33,34]. In those countries in which chewing betel quid and/or tobacco is highly popular including Taiwan [35-37], squamous cell carcinoma of the buccal mucosa is the most common oral cancer [38]. Despite the large amount of research into tumor cells and molecular biology, as well as recent advances in oncology and surgery, the rates of both mortality and morbidity in oral squamous cell carcinoma patients have remained unimproved [15].

In the search of anti-cancer chemicals, previous studies in our lab have shown the potential of several chemicals including a series of caffeic acid phenethyl ester-like compounds, methylantcinate A and triterpenoids derived from *Antrodia camphorata* to be promising anti-oral cancer drugs [16-18]. The activity of DB against cancer cells have been suggested or studied on murine leukemia, epidermal carcinoma, non-Hodgkin lymphoma, and non-small cell lung cancer cells [7-9,11,12]. We also report that DB has potential as a new therapeutic agent against human colorectal cancer and non-small cell lung cancer [10,13]. This study provided the first evidence that DB attenuates the growth of human oral cancer GNM and TSCCA cells, and might exert pro-apoptotic effects through a Bax-mediated mitochondrial apoptotic pathway and caspase cascade in time- and dose-dependent manners. Comparing with the aforementioned other chemicals, DB has the advantages on the accessibility and cost in addition to the prominent cytotoxicity against cancer cells. DB, one of the most abundant secondary metabolites of *M. anisopliae*[1], is secreted into the culture medium during growth. It is relatively cheap and easy to produce and purify DB from the medium rather than to synthesis it chemically. Moreover, as the safety of using DB *in vivo* has been demonstrated through the use of drug-related death rate, body weight loss, splenomegaly and pathological examination as parameters for evaluation in our previous report [10], results reported here may offer further impulse to the development of DB as a potential chemotherapeutic drug for oral and other cancers.

Apoptosis is a physiological cell death process to prevent individuals from tumor genesis or damage cancer cells. A recent study by Dornetshuber-Fleiss et al. [11] tested Dtx antiproliferative potential against a panel of human cancer cell lines and suggested that DB, in accordance with our previous study [10], being most effective in apoptosis induction in cancer cells of colorectal origin. However, the non-small cell lung cancer cell line A549 was remarkably resistant to it. In this study, the selective apoptotic cell death effects of oral cancer GNM and TSCCa cells nevertheless have been demonstrated with DB treatment. In addition, it was also suggested that 48 h treatment is necessary for DB to exert profound cell death, at least in clonogenicity assays. This is in accordance with our data, in our hands, the IC50s of DB treatment for 24 h are far greater than that of 48 and 72 h (Table 1).

## Conclusions

In summary, this is the first report on the anti-proliferation effect of DB in oral cancer cells. These data suggested that DB is capable to induce tumor specific growth inhibition in oral GNM and TSCCa cancer cells *via* Bax/Bcl-2-mediated intrinsic mitochondrial apoptotic pathway in time- and dose-dependent manners. Many lines of evidence demonstrate that Bax/Bcl-2-related proteins play an important role in either inhibition or promotion of apoptosis [39]. The Bcl-2 acts as an anti-apoptotic protein to prevent permeabilization of the outer mitochondrial membrane by inhibiting the action of the pro-apoptotic protein Bax [30]. Although the detailed molecular mechanism by which apoptosis is induced by this natural compound remains to investigated, and of course further studies including *in vivo* animal model are needed, the results reported here may offer further evidences to the development of DB as a potential complementary chemotherapeutic target for oral cancer complications.

## Abbreviations

DB: Destruxin B; DMSO: Dimethyl sulfoxide; Dtx: Destruxins; FACS: Fluorescence-activated cell sorter; FBS: Fetal bovine serum; FITC: Fluorescein isothiocyanate; GF: Gingival fibroblasts; GNM: Neck metastasis of gingival carcinoma; MTT: 3-(4,5-dimethylthiazol-2-yl)-2,5-diphenyl tetrazolium bromide, tetrazolium; NGS: Normal goat serum; PAGE: Polyacrylamide gel electrophoresis; PARP: Poly (ADP-ribose) polymerase; PI: Propidium iodide; PS: Phosphatidylserine; SDS: Sodium dodecyl sulfate; TBS: Tris buffered saline; TSCCa: Tongue squamous cell carcinoma.

## Competing interests

The authors declare that they have no competing interests.

## Authors’ contributions

HLR and CSP carried out the MTT, immunofluorescence, and western blotting studies, participated in data analysis and drafted the manuscript. LTM, TWY, and TCH carried out the flow cytometry and performed the statistical analysis. YCC and TYM conceived of the study, and participated in its design and coordination and helped to review the manuscript. All authors read and approved the final manuscript.

## Authors’ information

Yang CC is one of the editors of the following journals, including Cellular and Molecular Immunology (2010-present), Virus Genes (2010-present), The Open Infectious Disease Journal (SJR) (2008-present), Oriental Pharmacy and Experimental Medicine (2006-present), World Journal of AIDS (2011-present), World Journal of Virology (2011-present), and International Journal of Genuine Traditional Medicine, TANG (2011-present). Moreover, he is the executive editor of Chung Shan Medical Journal since 2012. Tzeng YM is the president of Biochemical Engineering Society of Taiwan (2010 ~ 2013). He is currently on the executive board of Asian Federation of Biotechnology (AFOB) and on the editorial board of Evidence-Based Complementary and Alternative Medicine, Biotechnology and Applied Biochemistry, and Journal of Bioscience and Bioengineering.

## Pre-publication history

The pre-publication history for this paper can be accessed here:

http://www.biomedcentral.com/1472-6882/14/207/prepub
